# Effect of current density on the microstructure and morphology of the electrodeposited nickel coatings

**DOI:** 10.3906/kim-2102-46

**Published:** 2021-10-19

**Authors:** Amel BOUKHOUIETE, Saliha BOUMENDJEL, Nour-El-Houda SOBHI

**Affiliations:** 1 Laboratory of Physical Metallurgy and Property of Materials, Badji-Mokhtar University, Annaba Algeria; 2 Hassiba Benbouali-Chlef University Algeria; 3 Water and Environment Science and Technology Laboratory, Mohamed Chérif Messaadia University, Souk-Ahras Algeria

**Keywords:** Nickel, electrodeposition, microstructure, morphology, current density

## Abstract

The aim of this research work was to study the effect of deposition current density on microstructure and surface morphology of electrodeposited nickel coatings. For this purpose, nickel deposits have been synthesized by direct current from Watts bath without additive, to limit the incorporation of pollutants resulting from surface adsorption or electro-activity of these compounds. Nickel deposits have been investigated by scanning electron microscopy and X-ray diffraction. Cyclic voltammetry was also used to gain information on the general behavior of the deposition. The optimum conditions of deposition were established and the influence of current density on the grain size, surface morphology, and crystal orientation was determined.

## 1. Introduction

Electroplating is a common electrochemical method, which improves the surface of various materials. The improvement relies on the formation of single or multilayer coatings on the surface of metals. Electroplated nickel is used extensively to enhance the utility, value, and appeal of manufactured products such as consumer goods. Other nickel coatings are used to improve the physical properties, the nickel coating serves the dual purpose of providing a bright, attractive finish as well as imparting improved corrosion resistance or other functional properties. Due to the excellent properties of these coatings, such as high hardness, excellent corrosion and wear resistance, self-lubricating properties and high thermal stability, they have good potential to replace based coatings of chrome. Their mechanism of deposition and optimization of process parameters must be well understood to produce better coatings with improved surface properties.

Many experimental works were published [1–5] to understand the relation between electrodeposition parameters and metallurgical states of the electrodeposits. The mechanism of nucleation and growth of Ni coatings was thoroughly described and, in most cases, illustrated from results coming from Watts baths [6, 7]. Properties of the deposited are strongly dependent on the experimental parameters of electrodeposition, such as bath composition, pH value, stirring solution, temperature, substrate, and applied current [8]. The current density plays an important role on the grain size and crystal structure of electrodeposited coating. The microstructure and grain size refinement in electrodeposited nickel can be controlled by several deposition parameters [8] like bath temperature, pH value, and applied current density. Generally, the grain size increases with temperature, whereas the grain size variation with current density is controversial. Therefore, this present work aims to study the effect of the current density on the microstructure of nickel deposits. In this paper, an additive-free watts bath is used in order to limit the incorporation of impurities. The thickness of the deposition layer of all the samples was 50µm, which is thick enough to attain a steady-state crystal growth condition. We also used a pure nickel substrate to avoid contamination of the bath. Special attention was devoted to avoid contamination of the bath.

## 2. Materials and methods

Nickel coatings were deposited on quite pure 99.5% polycrystalline nickel substrates with an average grain size of 165mm by direct current using a free-additive Watts bath. No additives were used. The composition of the conventional Watts bath is described in table 1. 

**Table 1:** Composition and plating conditions for Ni deposition from Watts bath.

NiSO4.7H2O NiCl2.6H2O Boric acid (H3BO3) pH value Temperature Stirring rate	300 g /L 45 g /L 45 g /L 4.5 60°C 200 rpm

A nickel sheet (50 cm^2^) of 99.99% high purity contained in a polypropylene anode bag was used as a soluble anode, and the exposed surface of the substrate was fixed at 2cm^2^. Before deposition, the nickel substrates were mechanically polished with silicon carbide paper of 600 to 4000 grits. Samples were then rinsed with ultrapure water and dried. Prior to characterization, all samples were stored in a desiccator. The coatings thickness was fixed at about 50µm by controlling the plating time. Gravimetric measurements were previously performed to check the cathodic efficiency and then to correct the deposit time to reach the required thickness.

Electroplating was performed using a conventional 3-electrodes devices contained in a 600 mL glass cell. Specific support was designed in order to keep constant the distance between each electrode during electroplating. Anode-cathode distance is kept constant at 3cm. The reference electrode was a saturated calomel electrode (SCE) inserted into the cell through a Luggin–Haber capillary tube. The distance between the capillary tube tip and the working electrode was 2mm.

The plating temperature was kept constant at 60 °C using a Julabo cryothermostat. The pH value was adjusted to a constant value of 4.5 by the addition of NaOH dilute solutions. The stirring rate was kept constant at 200 rpm during the electrodeposition.

Electrochemical experiments were performed using a VSP Biologic potentiostat/galvanostat coupled to a current amplifier VMP 3B-10 and driven by EC-lab software. 

Cyclic voltammetry was performed to determine the electrochemical reactions occurring on the surface of the substrate. The potential was scanned at a scan rate of 20 mV/s from –0.1 V versus the open circuit potential (OCP) until –1.8 V versus the reference electrode, and the reverse scan was stopped when the potential reaches the initial OCP value. Different experiments were performed to evaluate the reproducibility.

The surface morphology of the deposits was studied by scanning electron microscopy using a SEM-FEG Quanta Philips 200F. The deposit structures under various plating were characterized by X-ray diffraction using a Bruker D8 Advance diffractometer equipped with **a **Cu-Kα radiation. Diffractograms were recorded with a step size of 0.04° for 2θ ranging from 40 to 100°.

A microhardness tester (Shimatzu) was used for measuring hardness of coatings by performing Vickers indentation at a loading force of 200 g and holding time of 20 s.

## 3. Results

### 3.1. Cyclic voltammetry

**Figure 1:**
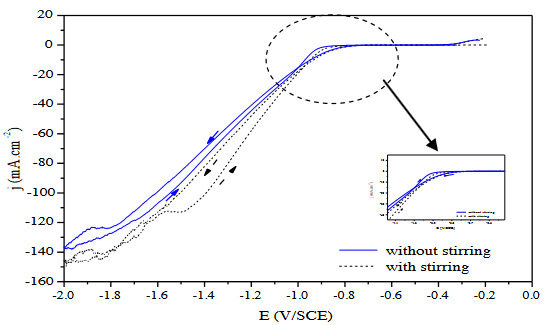
Voltammetric curves of nickel deposition at 20 mV/s from Watts bath without stirring compared with a stirred solution (200 rpm) at 20 °C.

Figure 1 presents the influence of the stirring condition on the electrodeposition mechanism of nickel from a conventional Watts bath at room temperature. A large current density plateau is obtained between –0.4 to –0.85 V/SCE corresponding to the reduction of the dissolved oxygen. Then, the current density increases due to the reduction of the nickel ions at the substrate interface. Without stirring and for high cathodic overpotential, a plateau of current density is observed at around 125 mA.cm^-2^, corresponding to the diffusional control of the growth of the nickel electrodeposited coating. When the solution is stirred, the current density plateau is slightly shifted toward higher current density values, approximately at 145mA.cm^-2^, favoring the nickel deposition.

During cathodic electrodeposition of nickel from Watts baths, two possible reactions can occur at the electrode:

(i) Deposition of nickel through the main reaction occurring at the electrode surface after decomplexation and/or 

dehydration of the nickel cations: Ni^2+^+2e^-^→ Ni (1).

(ii) During the electrodeposition process, hydrogen evolution reaction (HER) is produced at the cathode: 2H++ 2e- → H_2_ (2).

The increase of the current density at around –0.85V/SCE in the cathodic scan is due to the deposition of nickel and the hydrogen evolution reaction HER is characterized by identified reduction peak at more negative potentials around –1.7 V/SCE. Some authors suggested that the Volmer reaction of the HER could occur simultaneously with the nickel deposition [9], at cathodic potentials between–0.87 and –1.7 V/SCE, affecting the nucleation and growth mechanisms of the coatings.

Different studies have reported that nickel is first deposited in the presence of boric acid [10]. Boric acid may play the role of a catalyst by lowering the overvoltage for Ni deposition to the point where Ni is preferentially deposited instead of HER. This behavior is associated to the formation of a complex between Ni^2+^ ions and boric acid [11]. The overvoltage for Ni deposition in the presence of H_3_BO_3_ is low enough so that Ni is deposited at low current densities with H_ads_ being codeposited only at the highest current densities. Therefore, in the Watts bath, hydrogen adatoms are not deposited at low current densities [12], and the pH value at the metal-solution interface does not drastically change during the cathodic process. Effectively, boric acid was also reported to play a role of local buffer limiting the pH evolution at the substrate interface during the electrodeposition process. 

In the reverse scan where the potential was swept anodically, oxidation of nickel occurred but with an intensity much smaller than the one obtained during the nickel deposition. The oxidation of nickel could lead, when the electrolytic conditions become suitable, to the formation of the passive film composed of a mixture of NiO/Ni(OH)_2 _that limits the dissolution of the coating formed during the ingoing scan:

We can notice in Figure 1 that bath stirring has a very small effect on the reduction peak of nickel, indicating that the diffusion of Ni^2+^ is not the main determining reaction rate controlling the nickel deposition. The evolution of the diffusional plateau of metallic reduction at high cathodic overpotential is only slightly increased when stirring conditions are applied compared with an unstirred solution [13]. 

**Figure 2:**
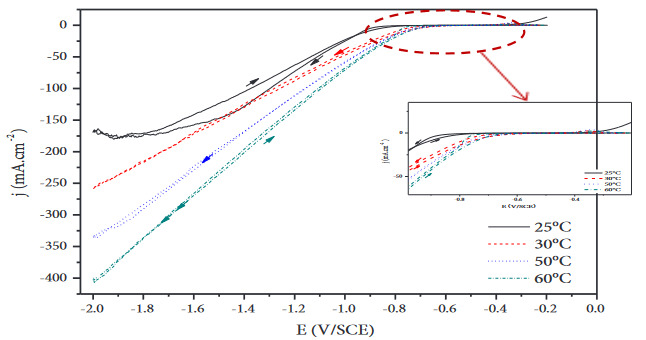
Influence of temperature on the cyclic voltammograms obtained on nickel in watts bath at a scan rate of 20 mV/s.

Figure 2 presents the influence of temperature on nickel deposition from Watts bath. At a temperature close to 25 °C, a diffusional control plateau is observed at high cathodic polarization. When the temperature is increased, the limit diffusional current density is shifted toward more cathodic potential, and the plateau is not more distinguishable in the range of explored potentials; the shape of the voltammetry curves does not change drastically with increasing the electrolyte temperature. Moreover, increasing the temperature leads to an increase in the current densities. The deposition of nickel is then favored with temperature. Most of studies found that the nickel deposition mechanism is very influenced by variation of the temperature, so high temperature improved the current efficiency of nickel [14] limiting the influence of the stirring conditions on the nucleation and growth of nickel deposits. Effectively, for temperatures up to 60 °C, the current density plateau associated to the diffusion control of nickel deposition progressively disappears, and the cathodic current densities considerably increase.

Metal ion activity also increases with increasing electrolyte temperature. A higher electrolyte temperature not only increases the solubility of the compounds of nickel but also increases the mobility of electrolyte components such as Ni^2+^ and H^+ ^[15]. As the temperature increases, the process may take place more easily and at a higher deposition rate [2]. The temperature of 60 °C was selected for the deposition of nickel, in agreement with the studies reported in the literature [15].

### 3.2. Synthesis of nickel coating

**Figure 3:**
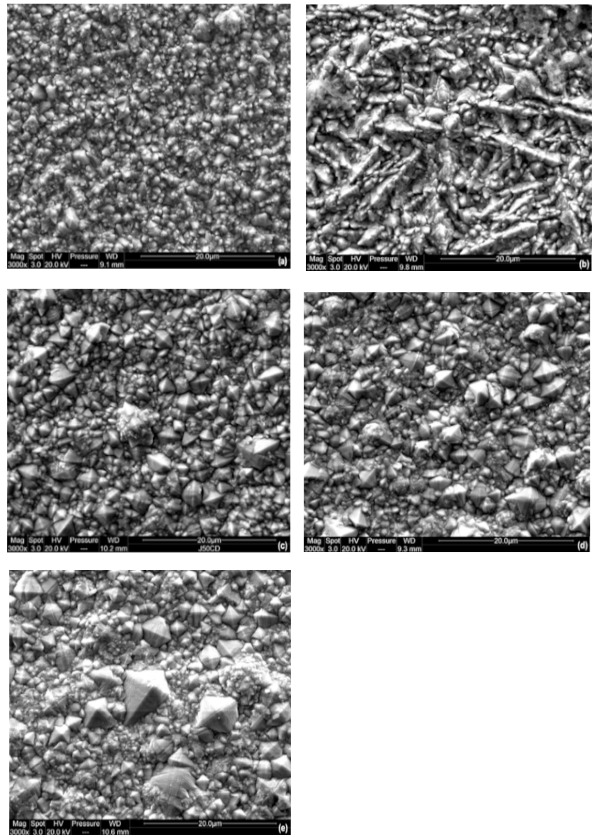
SEM images of nickel coatings electrodeposited at (a) 10, (b) 20, (c) 50, (d) 70, and (e) 100 mA/cm2.

Figure 3 presents the SEM observations of various nickel deposits prepared from Watts bath under direct current at different values of current densities from 10 to 100 mA/cm^2^. It can be seen that the nodule size of nickel deposits increases with increasing deposition current density. The smallest nodule size was obtained at a low current density of 10 mA/cm^2^ as observed in Figure 3a. Such phenomenon was also reported for nickel coatings synthesized from additive-free sulphamate bath [16]. Incorporation of impurities, especially light elements like hydrogen, oxygen, or nitrogen, was assigned as promoting the reduction of the nodule size. When the deposition current density is increased, the morphology of the coatings slightly evolves. The shape of the grains is not considerably changed except for the coatings deposited at 20 mA/cm^2 ^(Figure 3b) that present a typical morphology associated to the accumulation of truncated pyramids. For the highest value of current density (Figures 3c–3e), the surface morphology becomes heterogeneous containing large well-facetted pyramidal-shaped crystallites surrounded by unfaceted finer grains. The pyramidal growth is typical of a field-orientation texture, which exhibits preferential growth along the direction of the electric field [17]. The results revealed that the current density has an important influence on the surface morphology of electrodeposited Ni coatings. The coating deposited at 10 mA/cm^2 ^shows clearly smooth and compact surface with fine granules, as we can see in Figure 3**. **When the current density is increased, coarse nodules are formed, and a large distribution of nodules size is obtained. So, from these results, we can deduce that better uniformity and finer grain structure was obtained at low current densities. By increasing the deposition current density, grain size of the coating was increased. This can be explained by the fact that the electrodeposition at lower current densities takes time to reach the desirable thickness and, therefore, allows more time for the particles to be available at the cathode. This increases the chances that the particles will be incorporated homogeneously into the matrix, leading to the formation of harder surfaces due to dispersion hardening. Higher current densities increase the deposition rate but reduce the controllability of the deposition process. This causes rapid deposition, which poses a risk to the control of crystal growth and the uniformity of particle distribution within the matrix.

Table 2 presents the average sizes of the nodules deduced from the SEM surface observations and evaluated for different configurations at the same magnification using the intersection methods through the two image diagonals. The average size of nodules effectively increases with the current density. It can also be observed that large heterogeneities in nodule size occur at high current density, whereas the morphology of the coatings synthesized at lower current density is more homogeneous. Such evolutions were also observed with nickel-based coatings synthesized from sulphamate baths [16].

**Table 2:** Evolution of the average nodule size deduced from SEM observations of Ni based coatings deposited at different current density from a Watts bath.

j (mA/cm²)	dSEM (mm)
10	1.04 ± 0.39
20	1.15 ± 0.39
50	1.48 ± 0.74
70	1.51 ± 0.78
100	1.82 ± 1.38

**Figure 4:**
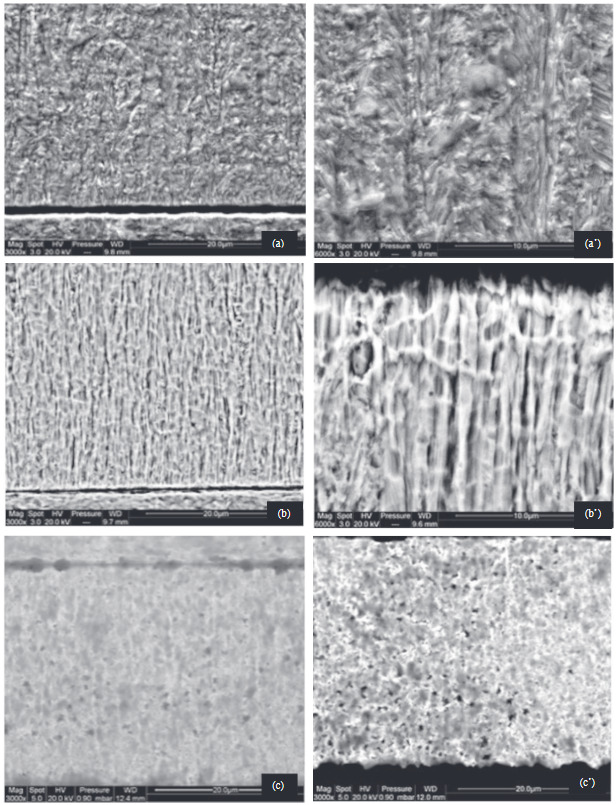
Cross-section view of nickel coatings electrodeposited at (a,a’) 10, (b,b’) 50, and (c,c’) 100 mA/cm2.

Cross-sectional observations (Figure 4) were made on the deposits obtained in continuous mode in order to complete the microstructural analysis. Several studies have been carried out in sections transverse in order to characterize the microstructure distribution within the matrix.

This study allowed us to highlight significant edge effects for the deposition made at high current densities. For low current densities (Figure 4a, Figure 4a’), very few edge effects were observed, and the coatings are uniform in thickness and exhibit very good bonding with the substrate. The deposits do not appear to have porosities. From Figure 4b and Figure 4c, we can see that, for the elaborate deposit with high current density, a morphology in the form of a column is observed. We observe several zones where the columns seem to widen as they grow. For low current densities, a fibrous morphology has been demonstrated. The fibers propagate through the coating perpendicular to the surface of the substrate. Links between some of these fibers are sometimes observed resulting in a change of direction from the direction of growth. Many authors have associated this type of morphology with a columnar structure [18].

**Figure 5:**
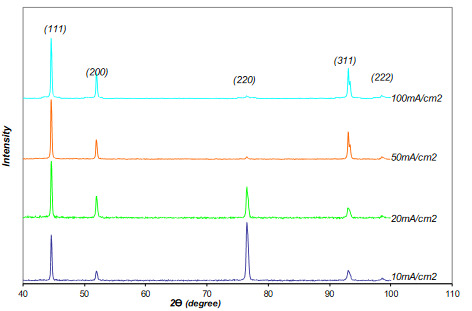
X-ray diffraction patterns of Ni coatings electrodeposited at different values of current densities from the Watts bath at 60 °C.

Figure 5 presents the X-ray diffraction (XDR) patterns of the electrodeposited Ni films deposited at direct current. The texture of thick deposits is completely independent of the crystal orientations of the substrate. As the thickness increases, the isolated clusters start to intersect and coalesce into a uniform film. Ultimately, the film becomes relatively uniform and flat; depending on the material mobility, the grain size may continue to change with the thickness as it grows. Thicker deposits exhibit various orientations, which depend solely on the electrodeposition conditions [3].

It can be seen from Figures 4 that the (220) peak becomes predominant at low current density (j= 10 mA/cm^2^). It was reported that this kind of structure appears preferentially at low current densities and for pH values between 1 and 5; the formation of this orientation can be attributed to the presence of H_ads _[3, 19]_, _which is adsorbed on the metallic surface and imposed this mode of growth [3]. When the current density increases, deposits have developed a (200) texture, and the intensity of the (220) peak is strongly reduced, whereas the obtained patterns seem to be close to a random orientation. This means that at low current density, the texture component of deposits is (220), but it changes to (200) at higher current densities. The change of preferred orientation with current density can be related to the existence of internal stress or porosity, both of which affect the hardness [20].

The relative texture coefficient N_(hkl)_ was used to determine the preferred crystalline orientations of the deposits and to evaluate their qualities [21] N_(hkl)_ is defined as:

N(hkl)=IF(hkl)IP(hkl)∑15(IF(hkl)IP(hkl)).100%

Where IF_(hkl)_ and I_P(hkl)_ are the diffraction intensities of the (hkl) plane measured in the diffractogram for the deposit and the standard Ni powder sample (JCPDS n°04-0850). A value of N_(hkl)_>1 indicates a preferred orientation of the (hkl) reflection compared with the random distribution of the grains. 

The texture index deduced from XRD patterns of the coatings synthesized at different values of current densities are listed in Table 3. At low current density, the texture index of the (220) is very high, approximately close to 4. When the current density increases, the (220) index decreases, and the indexes of the other microstructural orientations become close to 1. As the current densities decrease, grain refinement is observed associated with the evolution of the (100) texture towards a less marked (110) texture, in good agreement with published results [22]. The transition from (100) to (110) may be due to the residual strain around the interstitial H atoms. This behavior may support the postulate that the texture structure evolution of the transition metals is partly influenced by hydrogen evolution reaction (HER). For current densities higher than 50 mA/cm^2^, the texture index of the (311) orientation becomes abnormally high, whereas the (220) orientation is vanishing. The (311) texture was attributed to the presence of a colloidal dispersion of nickel hydroxide Ni(OH)_2 _[3]. Texture evolution is not so marked in the case of nickel coatings synthesized from Watts baths compared with the evolution observed for nickel coatings performed in free additive sulphamate bath [16]. 

**Table 4:** Vickers hardness (HV) of samples deposited from watts bath at different current density.

J (mA/cm²)	HV (Kg/mm2)	dSEM (mm)
10	355	1.04 ± 0.39
50	245	1.48 ± 0.74
100	250	1.82 ± 1.38

The intensity of (111) fiber orientation increases with an increase in the current density when it ranges from 20 to 100 mA/cm^2^. A quite random orientation is obtained with high current densities, probably due to the heterogeneous morphology and large distribution of nodule sizes. In direct current electrodeposition, a uniform distribution of nodule size is obtained, whereas coalescence of nodules in pyramidal-shape happens for higher current densities. So, coarse large pyramidal nodules are formed in a matrix composed of smaller nodules. Indeed, the pyramidal morphology has been observed by different authors, it being associated with coarse grains [23]. Moreover, it is often observed for high current densities [23] and associated with a texture (200), in agreement with our results. A common feature of the different baths used for nickel plating is that coarse-grain thick deposits are generally associated with (100) texture. This growth mode was referred to a free growth mode as it leads to ductile deposits with low internal stresses [24]. (100) texture is generally observed for high temperature and medium to high level of current density. Several authors by SEM observations have been able to show a refinement at low current densities [23] associated with texture formation (220). This is in accordance with our observations.

The data on microhardness of the nickel coatings are presented in Table 4. 

**Table 3:** Evolution of the relative texture index with the current density for coatings deposited in direct current.

			Texture index		
j(mA/cm)2	N(111)	N(200)	N(220)	N(311)	N(222)
10	0.635	0.418	3.995	0.823	1.243
20	0.833	0.800	2.229	0.823	1.417
50	0.965	0.708	1.368	1.344	1.041
100	0.947	0.924	0.267	2.068	0.906

As is seen, microhardness of deposit prepared at 10 mA/cm^2^ was very important compared to those obtained at higher density; this result is probably due to decrease in the grain size. In direct current, an increase in hardness could be observed in a Watts bath [25] and sulfamate bath when the current density decreases. Hardness increases with decreasing grain size. It has been illustrated [26] that the internal stress of deposits which relates to hardness increases as the grain size decreases. As the Ni thickness increases, the crystallite size of the film increases with smaller stress while the dislocation density decreases [27]. The porosity of nickel deposits, which reduces the hardness, has been proved to increase with an increase in current density [20]. The change of hardness is considered to relate to these three competing factors, i.e. grain size, internal stress, and porosity. Porosity that may reduce the hardness becomes more dominant, which lead to a decrease in hardness.

## 4. Conclusion 

In this study, electrodeposited nickel coatings were deposited on nickel substrates by direct current using Watts bath, without additives. The optimum conditions of deposition were established and the influence of current density on the grain size, surface morphology, and crystal orientation was determined. 

The results revealed that the shape, distribution, and nodule size are changed by varying the current density. This behavior may be explained by a change of the growth mechanism with current density. However, grain refinement was observed with decreasing deposition current density.

In conclusion, the current density has a remarkable influence on the surface morphology of electrodeposited nickel coatings.
